# Habitual nutrient intakes and postprandial glycaemic responses to glucose and orange juice among ethnically diverse adults in London: an exploratory cross-sectional analysis

**DOI:** 10.3389/fnut.2026.1884719

**Published:** 2026-09-07

**Authors:** Honglin Dong, Sophie Turner, A. F. M. Saiful Islam, Christian Reynolds, Swrajit Sarkar

**Affiliations:** 1 The School of Health and Medical Sciences, City St George’s, University of London, London, United Kingdom; 2 Centre for Food Policy, City St George’s, University of London, London, United Kingdom

**Keywords:** ethnicity, food diary, habitual nutrient intakes, postprandial glycaemic responses, type 2 diabetes mellitus

## Abstract

**Background:**

Postprandial glycaemic response (PPGR) is an important determinant of cardiometabolic risk; however, the association of habitual dietary nutrient intakes with PPGR across ethnically diverse populations remains poorly understood.

**Objectives:**

To compare differences in nutrient intakes among White Caucasian, Black African-Caribbean, and South Asian (SA) adults living in London, and to examine associations between habitual nutrient intakes and PPGRs to a standardized glucose drink and orange juice (OJ).

**Methods:**

Eighty-six healthy adults (White: *n* = 35, Black: *n* = 17, SA: *n* = 34) completed a 4-day estimated food diary and two postprandial glycaemic challenges following an overnight fast. Capillary blood glucose was measured at baseline (fasting) and every 30 min for 2 h. PPGR outcomes included total area under the curve (AUC) (0–120 min) and observed peak values (PVs) for each drink.

**Results:**

White participants reported significantly higher intakes of total energy, dietary fiber, calcium, magnesium, zinc, vitamin A, thiamine (B1), vitamin E, and folate than Black and/or SA participants. After adjusting for total energy intake, only dietary fiber, magnesium and folate intakes remained significantly different. SA participants exhibited significantly higher AUCs and PVs following consumption of both beverages than White participants or Black participants. In unadjusted regression analyses, nutrient intakes of dietary fiber, percentage energy from fat (fat %), and several micronutrients were associated with at least one PPGR outcome. After adjustment for age, body fat percentage, ethnicity, and total energy intake, dietary fiber, magnesium, thiamine (B1), and folate intakes were inversely associated with OJ-PV, whereas fat intake as a percentage of total energy was positively associated with OJ-AUC.

**Conclusion:**

These findings indicate that habitual dietary nutrient intakes were associated with PPGRs. Whether these dietary differences contribute to ethnic variation in PPGR requires further investigation.

## Introduction

Postprandial glycaemic response (PPGR) is defined as the change in blood glucose concentration following food or beverage consumption. High PPGR is a well-established risk factor for prediabetes, type 2 diabetes mellitus (T2DM), cardiovascular disease and all-cause mortality independent of fasting blood glucose and HbA1c (1). PPGR is influenced not only by the carbohydrate content of a meal, but also by physiological and behavioral factors including glucoregulatory hormones, gastric emptying, salivary or pancreatic amylase, diet, physical exercise, and sleep and circadian rhythm (2).

Diet is among the most important modifiable determinants of PPGR. Meal timing, composition, processing, and accompanying nutrients such as fiber, protein, and fat all influence PPGRs (3, 4). This body of research has largely focused on the acute, synchronous relationship between specific meal types and PPGR (2). Several studies have compared habitual nutrient intake profiles between healthy individuals and people with T2DM, identifying higher total energy intake, lower intakes of dietary fiber and several micronutrients among those with T2DM (5, 6). However, few studies have examined how habitual nutrient intakes are associated with PPGRs to a standardized meal or drink in a healthy and ethnically diverse population.

Ethnic disparities are well documented in the prevalence of T2DM. In the UK, the risk of developing T2DM is three times greater among Black African-Caribbean (Black) populations, and four times greater among South Asian (SA) populations than White Caucasians (White), with disease onset occurring at a younger age (7). Similar patterns have been reported in the United States (8). Although socioeconomic disadvantage, acculturation, lifestyle behaviors, and genetic susceptibility are recognized contributors to these disparities (9, 10), dietary patterns also vary substantially across ethnic groups because of differences in cultural traditions, socioeconomic circumstances, and food availability (11). Such dietary differences have been associated with a range of health outcomes, including cardiometabolic risk (12), blood lipid profiles (13), and gut microbiome composition (14). However, most previous studies have focused on dietary patterns or food groups rather than individual nutrient intakes. Although dietary pattern analyses capture the combined effects of foods consumed, they provide limited insight into the contributions of specific nutrients that may influence glucose metabolism through distinct physiological mechanisms. Consequently, it remains unclear whether habitual dietary nutrient intakes differ among ethnic groups and whether these nutrient intakes are associated with PPGRs.

Therefore, this study aimed to evaluate dietary nutrient intakes across White, Black, and SA ethnic groups, and to examine associations between habitual nutrient intakes and PPGR outcomes in an ethnically diverse population.

## Materials and methods

The present study is a cross-sectional secondary analysis of data collected from an acute randomized crossover repeated-measures trial investigating the ethnic differences in PPGR and its association with vitamin D status, the protocol for which has been published elsewhere (15). Although the parent study was designed to investigate ethnic differences in PPGRs following consumption of a glucose drink and orange juice, the current analysis is a secondary observational (cross-sectional) analysis that examines associations between habitual dietary nutrient intakes and PPGRs using prospectively collected dietary data. The 4-day estimated food diary was a pre-specified component of the original study protocol. The study was conducted between 1 November 2023 and 31 March 2025 at City St George’s, University of London. The study was approved by the Senate Research Ethics Committee at City St George’s, University of London (Ref: ETH22232000) and registered at ClinicalTrials.gov (NCT06241976) in November 2023. Informed consent was obtained electronically by an online screening questionnaire using Qualtrics (Qualtrics, Provo, US) and reconfirmed verbally in person at the first study visit.

The detailed study protocol, including participant recruitment, eligibility criteria, intervention procedures and sample size calculation, has been published previously (15). Only methods relevant to the present secondary analysis are described below.

### Participants

Inclusion criteria of the study were adults of 18–65 years who were residents in London, in general good health, and self-identified as White, SA, or Black. Exclusion criteria included diagnosed prediabetes or diabetes; gastrointestinal disease; BMI < 18.5 kg/m^2^; liver or kidney disease; other chronic illnesses (e.g., cardiovascular disease, cancer); blood-clotting disorders; alcohol intake >14 units/week; regular smoking (≥1 cigarette/day); pregnancy or lactation; and mixed ethnic background.

Participants were recruited from staff and students at City St George’s, University of London and through local community engagement activities with appropriate gatekeeper permissions and social media platforms (Meta, X, Instagram, and LinkedIn). Recruitment commenced at the same time as the trial registration (both in November 2023).

### Dietary nutrient intake assessment

A consecutive 4-day estimated food diary including 1 weekend day was collected from participants. All participants were trained on how to record their food diaries on their first visit. An example food diary was provided to participants. Both digital and hard copies were accepted. The participants were asked to record all foods and beverages consumed including supplements over four consecutive days (including one weekend day) with estimated portion sizes using household measures, package information or photographs where available. Participants were also encouraged to attach food photographs to improve the accuracy of portion size estimation and food identification if necessary. The food diaries were analysed using a nutrition analytical software, Nutritics version 6 (Nutritics Ltd., Dublin) to quantify the average daily energy and nutrient intakes. Nutritics software uses the UK Composition of Foods Integrated Dataset (CoFID) as the primary repository and supplemented with integrated global databases, such as the U.S. Department of Agriculture (USDA) Branded Food Products Database, to accurately capture ethnically diverse ingredients. Traditional recipes and brand-specific items absent from standard registries were input via the Nutritics Custom Recipe Builder or used the closest matching food items or standard recipes available within the software. Nutrient intakes from dietary supplements were included only if reported in the food diary.

An experienced research assistant (ST) analysed all food diaries and discussed any unsure food items with the principal investigator (HD). Dietary analyses included nearly all nutrients generated from Nutritics, including total energy intake, percentage (%) energy from macronutrients (carbohydrate, protein, fat, saturate fat, free sugar), dietary fiber, minerals (calcium, magnesium, zinc, iron, iodine, selenium) and vitamins (vitamin A, B1, B2, B3, B6, B12, C, E, K1 and folate). Vitamin D intake was not included due to its main source being from sunlight exposure instead of diet. No individual micronutrients were pre-specified as primary exposures because this was an exploratory secondary analysis.

To minimize the impact of severe misreporting, daily dietary logs were screened using standard epidemiological energy intake cut-offs. Individual records with an average intake of <500 kcal/day or >3,500 kcal/day were classified as biologically implausible and excluded from the final data analysis (16). The plausibility of reported energy intake was assessed using study-specific Goldberg energy intake (EI)-to-basal metabolic rate (BMR) ratio (EI:BMR) based on a 4-day dietary recording period, sample size (*n* = 86) and an assumed physical activity level of 1.55 (we did not collect this information), to identify potential under-reporters (EI: BMR < 1.03) and over-reporters (EI:BMR > 2.33) (17). The proportions of under-reporters, plausible reporters, and over-reporters were compared among ethnic groups using Pearson’s chi-square test. Pearson’s correlation analysis was performed to examine the associations of BMI and body fat percentage (BF%) with the EI:BMR. Results are presented as the correlation coefficient (*r*) and *P*-value.

### Postprandial glycaemic challenges

Participants consumed two different beverages on two occasions separated by at least a 48-h washout period following an overnight fast (minimum of 10 h). On each occasion, participants consumed within 5 min either a glucose drink - 75 g dextrose (Bulk, Essex, UK) in 300 mL water (281 kcal), consistent with the oral glucose tolerance test (OGTT) practice (18); or 100% pure squeezed orange juice (Tesco, UK), 300 mL (129 kcal; 30 g sugar; 0.3 g fiber; 1.8 g protein; 90 mg vitamin C) to represent a commonly consumed sugar-containing beverage under ecologically relevant conditions. Capillary blood glucose was measured by HemoCue Glucose 201+ analyser (Health-care Equipment and Supplies, Surrey, UK) via finger-prick sampling at baseline (0 min) and at 30 min intervals over a 2-h postprandial period. The order of the two beverages was randomized using the Excel RANDBETWEEN function to allocate the first visit (1 = glucose; 0 = orange juice). Neither researchers nor participants were blind to the treatment beverages because of obvious sensory differences.

During the 2-h postprandial period, participants remained sedentary and refrained from eating, drinking, and engaging in physical activity. To minimize intra-individual variability, participants were asked to consume similar evening meals before visits, avoid alcohol and strenuous activity, and maintain consistent sleep prior to their visit.

Participant characteristics including age, gender, ethnicity and education levels (GCSE or equivalent, A level or equivalent, undergraduate or diploma, postgraduate or above) were collected and the body height, body weight and body fat percentage (BF%) were measured by a stadiometer and a dual frequency bioelectrical impedance device, TANITA DC-360 P (Tanita, Tokyo, Japan), respectively.

### PPGR outcome variables

Postprandial glycaemic responses to the above two drink consumptions were presented as the area under the curve (AUC) and observed peak values (PVs). Four postprandial glycaemic response (PPGR) outcomes were derived for each participant including G-AUC and G-PV after glucose (G) drink consumption, and OJ-AUC and OJ-PV after orange juice (OJ) consumption. The AUC was calculated via trapezoidal method (19).

### Statistical analysis

The sample size of the study was based on the primary outcome measures, PPGRs, with assumed effect size 0.25 for between subject factors, five repeated glucose measurements, three ethnic groups, 80% power, and α = 0.05 using *a priori* sample size calculations (G*Power v3.1.9.7). A total of 96 participants were needed and we recruited 113 participants. Only 87 participants provided food diaries, among which 2 were excluded due to low energy intake (<800 kcal/day). Therefore, data from 85 participants were included for statistical analysis.

Categorical variables were presented as frequency (n) and percentage (%). Continuous variables were presented as mean ± standard deviation (SD) or median (interquartile range, IQR) if data were not normally distributed. Data normality was assessed using the Shapiro-Wilk test. Between-group differences in continuous variables were assessed using one-way ANOVA or the Kruskal-Wallis test, as appropriate according to data distribution. *Post hoc* pairwise comparisons were adjusted using the Bonferroni method. The sex distribution between groups was examined using Pearson’s chi-square test. The nutrient intakes were presented as unadjusted (per day) and adjusted for total energy intake as per 1000 kcal/day. Only the nutrient intakes that showed significant differences between ethnic groups were reported in the results.

Associations between individual nutrient daily intakes (not energy-adjusted) and each PPGR outcome were assessed by simple (unadjusted) and multiple linear regression (adjusted) using a standard adjustment approach. Regression assumptions were assessed for all linear regression models. Multicollinearity was assessed using variance inflation factors (VIFs), with a maximum VIF of 2.009 across the models. Potentially influential observations were assessed using Cook’s distance, with a maximum value of 0.313. Residual plots showed random scatter around zero, and residuals were approximately normally distributed. Overall, the diagnostic assessments indicated no substantial violations of the assumptions of linear regression. Due to the exploratory nature of the study and the large number of nutrient variables, only associations significant in either unadjusted model or adjusted models are presented. Model 1 was adjusted for age, BF%, and ethnicity, while Model 2 was further adjusted for total daily energy intake such that the results would indicate whether the composition of the diet matters independent of how much food was consumed. Dummy coding was used to transform categorical ethnicity data into numerical binary variables for the multiple linear regression analysis, and the White group served as the reference category. Because BMI and BF% were highly correlated (*r* = 0.750, *P* < 0.001 based on the data of the current study), BF% was retained as the covariate representing adiposity. Regression coefficients (B), standard errors (SE), t-statistics, *P*-values and 95% confidence interval (95% CI) were reported.

As this was an exploratory analysis intended to generate hypotheses, correction for multiple testing was not performed. Statistical significance was set at *P* < 0.05. All analyses were conducted using SPSS (version 28, IBM Corp., Armonk, NY, USA).

## Results

### Participant characteristics

A total of 87 participants provided food diaries in the study. One participant was excluded from the analysis due to an incomplete food diary, which reported an energy intake of 360 kcal/day. The final analysis therefore included 86 participants: White (*n* = 35), Black (*n* = 17), and SA (*n* = 34).

Table 1 shows the characteristics of the participants. The median age was 31 (24.0, 40.5) years; however, SA participants were significantly younger [26 (20.0, 33.0) years] than both White [36.0 (29.0, 40.0) years, *P* = 0.003] and Black participants [35 (27.0, 46.0) years, *P* = 0.044]. The majority of the participants (81.4%, *n* = 70) were female (three participants preferred not to disclose their sex, therefore categorized as “unknown”), and there was no significant difference in sex composition between ethnic groups (*P* = 0.439). The median BMI was 26.0 (22.8, 27.8) kg/m^2^ for all participants, and Black participants had a significantly higher BMI [27.6 (25.9, 33.2) kg/m^2^] than SA participants [24.2 (21.8, 26.8) kg/m^2^, *P* = 0.004]. There was no significant difference in education levels among ethnic groups (*P* = 0.176).

**TABLE 1:** Characteristics of the participants.

Characteristics		All participants	White	Black	SA	*P*	Pairwise comparisons
Sample size [*n* (%)]		86 (100%)	35 (41.2%)	17 (20.0%)	34 (38.8%)	–	–
Age (year)		31 (24.0–40.5)	36.0 (29.0–40.0)	35 (27.0–46.0)	26 (20.0–33.0)	**0.005**	SA vs. Black, ***P* = 0.044**
							SA vs. White, ***P* = 0.003**
Sex	Female	70 (81.4%)	26 (74.3%)	15 (88.2%)	29 (85.3%)	0.439	–
	Male	13 (15.1%)	7 (20.0%)	1 (5.9%)	5 (14.7%)		
	Unknown	3 (3.5%)	2 (5.7%)	1 (5.9%)	0		
BMI (kg/m^2^)		26.0 (22.8–27.8)	26.1 (23.0–28.3)	27.6 (25.9–33.2)	24.2 (21.8–26.8)	**0.005**	Black vs. SA, ***P* = 0.004**
BF%		30.9 ± 7.9	30.6 ± 7.9	34.6 ± 8.0	29.3 ± 7.4	0.069	–
Education							
	GCSE	3 (3.5%)	2 (5.7%)	1 (5.9%)	0	0.176	–
	A level	18 (21.2%)	4 (11.4%)	4 (23.5%)	10 (29.4%)		
	Undergraduate	28 (32.9%)	9 (25.7%)	7 (41.2%)	12 (35.3%)		
	Postgraduate	36 (42.4%)	20 (57.1%)	5 (29.4%)	12 (35.3%)		

Statistical methods: categorical variables - Pearson chi-square test; continuous variables - Kruskal-Wallis test with Bonferroni correction [presented as median (IQR)] or one-way ANOVA with Bonferroni correction (presented as mean ± SD). BF%, body fat percentage; BMI, body mass index; IQR, interquartile range; SA, South Asian. All significant *P*-values were highlighted in bold and *P* represents *P*-value.

### Nutrient intakes across ethnic groups

Significant between-group differences were observed in some nutrient intakes (unadjusted or adjusted) (Table 2) (only the significant results were reported). White participants reported the highest total energy intake, median 2064 kcal/day, which was significantly greater than SA (median 1613.5 kcal/day, *P* = 0.012) participants. White participants also consumed significantly more dietary fiber, calcium, iron, magnesium, vitamin A, B1, B2, and folate (B9) compared with both Black and SA participants. Zinc, iodine and vitamin E intakes were significantly higher in White compared with Black participants. There was no significant difference in nutrient intakes between Black and SA participants.

**TABLE 2:** Nutrient intakes among ***White*** (***n*** = 35), Black (***n*** = 17) and South Asian (***n*** = 34) groups.

Nutrient	Ethnicity	Unadjusted (per day)			Energy-adjusted (per 1000 kcal/day)		
		Mean ± SD or Median (IQR)	*P*	Pairwise comparisons	Mean ± SD or Median (IQR)	*P*	Pairwise comparisons
Energy (kcal)	White	2064.1 (1573.6, 2569.4)	0.008	White vs. SA, ***P* = 0.012** White vs. Black, *P* = 0.071	–	–	–
	Black	1603.9 (1553.1, 1916.2)			–		
	SA	1613.5 (1392.1, 1932.9)			–		
	Total	1734.9 (1475.4, 2149.4)					
Fiber (g)	White	23.1 (17.6, 29.5)	<0.001	White vs. Black, ***P* < 0.001** White vs. SA, ***P* = 0.011**	12.3 (8.6, 16.3)	**0.043**	White vs. Black, ***P* = 0.037** White vs. SA, *P* = 0.738
	Black	14.7 (11.2, 17.3)			8.6 (6.9, 11.5)		
	SA	17.6 (14.2, 23.0)			10.4 (8.9, 13.5)		
	Total	18.3 (14.9, 25.3)			10.5 (8.1, 14.7)		–
Calcium (mg)	White	739.7 (634.0, 984.2)	0.002	White vs. Black, ***P* = 0.005** White vs. SA, ***P* = 0.021**	415.7 ± 132.2	0.092	–
	Black	505.4 (412.7, 765.2)			336.8 ± 116.1		
	SA	581.5 (467.0, 780.2)			380.0 ± 113.2		
	Total	648.4 (482.0, 856.8)			386.0 ± 124.0		–
Iron (mg)	White	10.8 (8.8, 15.1)	0.009	White vs. Black, ***P* = 0.035** White vs. SA, ***P* = 0.029**	5.3 (4.4, 7.0)	0.603	–
	Black	8.6 (5.7, 13.1)			5.5 (3.9, 6.8)		
	SA	9.2 (7.0, 10.7)			5.2 (4.3, 6.1)		
	Total	9.7			5.3 (4.3, 6.5)		
		(7.5, 12.7)					–
Zinc (mg)	White	7.6 (6.4, 10.6)	0.022	White vs. SA, ***P* = 0.027**	4.0 (3.2, 4.9)	0.919	–
	Black	5.9 (4.9, 9.4)			3.7 (3.2, 5.4)		
	SA	6.7 (5.0, 8.2)			4.1 (3.2, 4.7)		
	Total	6.9 (5.5, 9.0)			4.0 (3.2, 4.7)		
Magnesium (mg)	White	310.8 (267.1, 399.3)	<0.001	White vs. Black, ***P* < 0.001** White vs. SA, ***P* < 0.001**	171.8 (140.8, 204.3)	**0.001**	White vs. Black, ***P* = 0.002** White vs. SA, ***P* = 0.030**
	Black	190.4 (142.1, 271.1)			126.3 (92.4, 146.9)		
	SA	208.7 (176.3, 296.5)			143.0 (121.9, 161.1)		
	Total	259.5 (192.6, 350.2)			147.5 (121.9, 179.6)		
Iodine (μg)	White	112.1 (67.7, 175.1)	0.021	White vs. Black, ***P* = 0.016**	54.8 (35.8, 81.5)	0.115	–
	Black	63.9 (36.0, 75.0)			41.7 (22.0, 46.4)		
	SA	88.7 (46.1, 128.4)			51.9 (31.1, 83.8)		
	Total	89.1 (47.0, 128.4)			49.6 (33.4, 79.3)		
Vitamin A (μg)	White	868.0 (499.3, 1271.8)	0.005	White vs. Black, ***P* = 0.020** White vs. SA, ***P* = 0.018**	430.4 (238.3, 607.9)	0.235	–
	Black	503.5 (315.7, 718.3)			333.6 (201.2, 486.8)		
	SA	495.6 (300.7, 922.1)			304.3 (187.7, 628.9)		
	Total	633.0 (385.7, 969.7)			358.5 (216.9, 558.3)		
Thiamine (B1) (mg)	White	1.4 (1.1, 1.8)	<0.001	White vs. Black, ***P* = 0.022** White vs. SA, ***P* = 0.001**	0.7 (0.6, 0.9)	0.240	–
	Black	1.1 (0.8, 1.2)			0.7 (0.5, 0.8)		
	SA	1.0 (0.8, 1.4)			0.6 (0.5, 0.7)		
	Total	1.2 (0.9, 1.5)			0.7 (0.5, 0.8)		
Riboflavin (B2) (mg)	White	1.5 (1.3, 1.9)	0.002	White vs. Black, ***P* = 0.017** White vs. SA, ***P* = 0.008**	0.7 (0.6, 0.9)	0.301	–
	Black	1.0 (0.8, 1.4)			0.6 (0.5, 0.8)		
	SA	1.1 (0.9, 1.5)			0.7 (0.5, 0.9)		
	Total	1.3 (0.9, 1.6)			0.7 (0.6, 0.9)		
Vitamin E (mg)	White	9.2 (6.7, 14.7)	0.042	White vs. Black, ***P* = 0.047**	5.2 (3.6, 6.3)	0.352	–
	Black	6.3 (5.0, 10.0)			3.6 (3.3, 5.7)		
	SA	8.5 (5.9, 10.4)			5.0 (3.8, 6.3)		
	Total	8.5 (6.2, 11.7)			4.9 (3.5, 6.1)		
Folate (μg)	White	283.8 (212.3, 378.8)	<0.001	White vs. Black, ***P* < 0.001** White vs. SA, ***P* = 0.002**	150.9 ± 54.2	**0.005**	White vs. Black, MD 46.8, 95% CI (11.1, 82.5), ***P* = 0.006** White vs. SA, MD 26.8, 95% CI (−2.2, 55.8), *P* = 0.081
	Black	172.7 (138.6, 232.3)			104.1 ± 40.7		
	SA	203.4 (125.9, 261.1)			124.1 ± 48.0		
	Total	223.7 (167.8, 301.7)			131.1 ± 52.0		

Statistical methods: categorical variables - Pearson chi-square test; continuous variables - Kruskal-Wallis test with Bonferroni correction [presented as median (IQR)] or one-way ANOVA with Bonferroni correction (presented as mean ± SD). BF%, body fat percentage; BMI, body mass index; IQR, interquartile range; SA, South Asian. All significant *P*-values were highlighted in bold and *P* represents *P*-value.

After nutrient intakes were adjusted for total energy intake, only dietary fiber, magnesium and folate intakes remained significantly different between ethnic groups (Table 2). White participants showed significantly higher energy-adjusted dietary fiber and folate intake than Black participants only and had significantly higher energy-adjusted magnesium intake than both Black and SA groups (*P* = 0.002 and *P* = 0.030, respectively).

### PPGRs to glucose drink and orange juice consumption

Fasting glucose concentrations and OGTT results were within the normal range for all participants (≤5.5 mmol/L and ≤7.8 mmol/L at 2 h, respectively) (20). There was no significant difference in the fasting blood glucose concentrations between ethnic groups before either glucose drink or orange juice consumption (Table 3). However, the AUCs after both glucose drink (G-AUC) and orange juice consumption (OJ-AUC) were significantly higher in SA than White participants, while the PVs after both glucose drink (G-PV) and orange juice consumption (OJ-PV) were significantly higher in SA than those of White and Black participants, respectively (Table 3). One participant did not take the glucose drink due to withdrawal.

**TABLE 3:** Comparisons of AUCs (0–120 min) and PVs between ethnic groups.

Postprandial glycaemic variables	All participants	White	Black	SA	*P*	Pairwise comparisons
Glucose drink	*n* = 84	*n* = 35	*n* = 16	*n* = 33	–	–
G-0 (mmol/L)	4.60 ± 0.50	4.51 ± 0.49	4.67 ± 0.58	4.67 ± 0.45	0.346	–
G-PV (mmol/L)	7.58 ± 1.32	7.08 ± 1.15	7.49 ± 1.70	8.15 ± 1.06	<**0.001**	SA vs. White, MD 1.07, 95% CI (0.34, 1.81), ***P* = 0.001**
G-AUC (mmol⋅min/L)	757.6 ± 122.0	713.6 ± 110.9	749.3 ± 156.1	808.4 ± 96.5	<**0.001**	SA vs. White, MD 94.84, 95% CI (26.28, 163.39), ***P* = 0.003**
Orange juice	*n* = 85	*n* = 35	*n* = 17	*n* = 33	–	–
OJ-0 (mmol/L)	4.55 ± 0.51	4.48 ± 0.46	4.58 ± 0.56	4.59 ± 0.54	0.637	–
OJ-PV (mmol/L)	6.46 ± 0.94	6.41 ± 1.00	5.94 ± 1.00	6.78 ± 0.72	**0.009**	SA vs. Black, MD 0.84, 95% CI (0.19, 1.49), ***P* = 0.007**
OJ-AUC (mmol⋅min/L)	588.2 ± 76.2	571.2 ± 72.9	566.6 ± 81.7	617.5 ± 69.4	<**0.001**	SA vs. White, MD 46.34, 95% CI (2.82, 89.86), ***P* = 0.033**

Data were presented as mean ± SD. MD, mean difference; 95% CI, 95% confidence interval. One-way ANOVA was used adjusted by Bonferroni correction. G-0, fasting glucose concentration before consuming glucose drink; G-AUC, the area under the curve following glucose drink consumption; G-PV, observed peak value following glucose drink consumption; OJ-0, fasting glucose concentration before consuming orange juice; OJ-AUC, the area under the curve following orange juice consumption; OJ-PV, observed peak value following orange juice consumption; SA, South Asian. All significant *P*-values were highlighted in bold and *P* represents *P*-value.

### Associations between nutrient intakes and PPGRs

Table 4 presents the linear regression coefficients for associations between dietary nutrient intakes and PPGR outcomes across three models (Unadjusted, Model 1 and Model 2). Only the significant associations in the unadjusted or adjusted models were shown in the results.

**TABLE 4:** Association of nutrient intakes with PPGRs represented as AUCs and PVs.

Nutrients	Mode	G-AUC (mmol⋅min/L)					G-PV (mmol/L)					OJ-AUC (mmol⋅min/L)					OJ-PV (mmol/L)				
		B	SE	*t*	*P*	95% CI	B	SE	*t*	*P*	95% CI	B	SE	*t*	*P*	95% CI	B	SE	*t*	*P*	95% CI
Fat (% total energy)	Unadjusted	0.860	2.256	0.381	0.704	−3.628, 5.348	0.000	0.024	0.005	0.996	−0.048, 0.049	2.825	1.370	2.062	**0.042**	0.100, 5.550	0.031	0.017	1.811	0.074	−0.003, 0.065
	Model 1	0.984	2.097	0.469	0.640	−3.190, 5.158	0.002	0.023	0.105	0.916	−0.043, 0.048	2.834	1.314	2.157	**0.034**	0.219, 5.448	0.030	0.017	1.821	0.072	−0.003, 0.063
	Model 2	1.051	2.121	0.496	0.621	−3.171, 5.274	0.004,	0.023	0.175	0.862	−0.042, 0.050	3.024	1.316	2.299	**0.024**	0.406, 5.643	0.032	0.017	1.905	0.060	−0.001, 0.065
Fibre (g/day)	Unadjusted	−3.125	1.191	−2.624	**0.010**	−5.495, −0.756	−0.040	0.013	−3.129	**0.002**	−0.065, −0.014	−2.071	0.739	−2.801	**0.006**	−3.541, −0.601	−0.023	0.009	−2.460	**0.016**	−0.041, −0.004
	Model 1	−1.846	1.323	−1.395	0.167	−4.480, 0.787	−0.024	0.014	−1.706	0.092	−0.050, 0.004	−1.775	0.843	−2.106	**0.038**	−3.452, −0.098	−0.030	0.010	−2.874	**0.005**	−0.050, −0.009
	Model 2	−2.048	1.457	−1.406	0.164	−4.950, 0.853	−0.024	0.016	−1.561	0.123	−0.056, 0.007	−1.689	0.928	−1.820	0.073	−3.537, 0.158	−0.032	0.011	−2.819	**0.006**	−0.055, −0.009
Magnesium (mg/day)	Unadjusted	−0.252	0.107	−2.265	**0.020**	−0.465, −0.040	−0.003	0.001	−2.812	**0.006**	−0.005, −0.001	−0.177	0.066	−2.681	**0.009**	−0.308, −0.046	−0.001	0.001	−1.701	0.093	−0.003, 0
	Model 1	−0.125	0.120	−1.038	0.302	−0.363, 0.114	−0.002	0.001	−1.269	0.208	−0.004, 0.001	−0.156	0.076	−2.048	0.044	−0.307, −0.004	−0.002	0.001	−2.043	**0.044**	−0.004, 0
	Model 2	−0.165	0.150	−1.098	0.275	−0.464, 0.134	−0.002	0.002	−1.083	0.282	−0.005, 0.001	−0.167	0.095	−1.749	0.084	−0.357, 0.023	−0.002	0.001	−2.027	**0.046**	−0.005, 0
Vitamin A (μg/day)	Unadjusted	−0.035	0.023	−1.522	0.132	−0.080, 0.011	0	0	−1.325	0.189	−0.001, 0	−0.030	0.014	−2.142	**0.035**	−0.058, −0.002	0	0	−1.498	0.138	−0.001, 0
	Model 1	−0.018	0.023	−0.771	0.443	−0.064, 0.028	0	0	−0.281	0.779	−0.001, 0	−0.026	0.015	−1.759	0.082	−0.055, 0.003	0	0	−1.775	0.080	−0.001, 0
	Model 2	−0.017	0.024	−0.728	0.469	−0.065, 0.030	0	0	−0.138	0.891	−0.001, 0	−0.024	0.015	−1.560	0.123	−0.054, 0.006	0	0	−1.656	0.102	−0.001, 0
Vitamin B1 (mg/day)	Unadjusted	−42.301	18.471	−2.290	**0.025**	−79.040, −5.563	−0.529	0.197	−2.686	**0.009**	−0.921, −0.137	−32.935	11.334	−2.906	**0.005**	−55.474, −10.396	−0.438	0.139	−3.150	**0.002**	−0.714, −0.161
	Model 1	−16.642	19.251	−0.865	0.390	−54.960, 21.675	−0.241	0.207	−1.160	0.250	−0.653, 0.172	−22.661	12.232	−1.853	0.068	−47.003, 1.681	−0.427	0.149	−2.870	**0.005**	−0.723, −0.131
	Model 2	−17.695	21.335	−0.829	0.409	−60.170, 24.779	−0.222	0.230	−0.967	0.337	−0.680, 0.235	−20.881	13.526	−1.544	0.127	−47.803, 6.042	−0.463	0.164	−2.820	**0.006**	−0.790, −0.136
Vitamin B2 (mg/day)	Unadjusted	−27.412	15.431	−1.776	0.079	−58.104, 3.279	−0.340	0.165	−2.056	**0.043**	−0.669, −0.011	−24.287	9.442	−2.572	**0.012**	−43.064, −5.511	−0.250	0.118	−2.115	**0.037**	−0.484, −0.015
	Model 1	−9.087	15.770	−0.576	0.566	−40.476, 22.302	−0.113	0.170	−0.666	0.507	−0.453, 0.226	−16.700	10.036	−1.664	0.100	−36.672, 3.273	−0.230	0.125	−1.840	0.069	−0.479, −0.019
	Model 2	−8.913	17.321	−0.515	0.608	−43.395, 25.570	−0.082	0.187	−0.438	0.663	−0.454, 0.290	−14.839	11.016	−1.347	0.182	−36.765, 7.088	−0.233	0.137	−1.605	0.093	−0.507, 0.040
Folate (μg/day)	Unadjusted	−0.260	0.100	−2.595	**0.011**	−0.459, −0.061	−0.003	0.001	−2.926	**0.004**	−0.005, −0.001	−0.159	0.063	−2.545	**0.013**	−0.284, −0.035	−0.002	0.001	−2.196	**0.031**	−0.003, 0
	Model 1	−0.137	0.112	−1.228	0.223	−0.407, 0.096	−0.002	0.001	−1.376	0.173	−0.004, 0.001	−0.128	0.072	−1.78	0.078	−0.271, 0.015	−0.002	0.001	−2.565	**0.012**	−0.004, −0.001
	Model 2	−0.155	0.126	−1.183	0.240	−0.404, 0.103	−0.002	0.001	−1.200	0.234	−0.004, 0.001	−0.118	0.081	−1.457	0.149	−0.278, 0.043	−0.002	0.001	−2.506	**0.014**	−0.004, −0.001
Vitamin E (mg/day)	Unadjusted	−5.197	2.834	−1.833	0.070	−10.834, 0.441	−0.071	0.030	−2.338	**0.022**	−0.131, −0.011	−1.349	1.793	−0.752	0.454	−4.915, 2.217	−0.018	0.022	−0.810	0.420	−0.062, 0.026
	Model 1	−3.428	2.841	−1.207	0.231	−9.082, 2.226	−0.045	0.031	−1.475	0.144	−0.106, 0.016	−0.528	1.849	−0.286	0.776	−4.207, 3.151	−0.024	0.023	−1.029	0.307	−0.069, 0.022
	Model 2	−4.318	3.407	−1.268	0.209	−11.101, 2.464	−0.049	0.037	−1.321	0.190	−0.122, 0.025	0.746	2.207	0.338	0.736	−3.648, 5.139	−0.022	0.028	−0.782	0.436	−0.077, 0.033

B, unstandardized coefficient; SE, standard error of B; *t*, *t*-*statistic*; 95% CI, 95% confidence interval. Simple linear regression (unadjusted) and multiple linear regression adjusted for age, BF% and ethnicity (Model 1) and further adjusted for total energy intake (Model 2). G-AUC, the area under the curve after glucose drink consumption; G-PV, observed peak value after glucose drink consumption; OJ-AUC, the area under the curve after orange juice consumption; OJ-PV, observed peak value after orange juice consumption. All significant *P*-values were highlighted in bold and *P* represents *P*-value.

Dietary fiber, fat (%), magnesium, vitamin A, B1, B2, E, and folate intakes were significantly associated with at least one of the PPGR outcomes (GPV, GAUC, OJPV and OJAUC) in the unadjusted model. After adjusting for age, BF% and ethnicity (Model 1), vitamin A and E became insignificant with any PPGR outcomes. After further adjusting for total energy intake (Model 2), only dietary fiber, magnesium, vitamin B1 and folate intakes had significant inverse associations with OJ-PV, while fat (%) showed a significant positive association with OJ-AUC. No significant association was observed with the glucose drink challenge.

### Under-report and over-report of energy intake for the food diaries

Table 5 shows that 21 participants (24.4%) were classified as under-reporters, while the majority (74.4%, *n* = 64) were classified as plausible reporters. Only one participant (1.2%) was classified as an over-reporter. There was no significant difference in the distribution of energy intake reporting status among the ethnic groups (*P* = 0.668). Pearson’s correlation analysis demonstrated significant inverse correlations between BMI and EI:BMR (*r* = −0.392, *P* < 0.001) and between BF% and EI:BMR (*r* = −0.327, *P* = 0.002).

**TABLE 5:** Plausibility of energy intake report among ethnic groups.

Plausibility	Total	White	Black	South Asian	*P*
Sample size	86 (100%)	35 (100%)	17 (100%)	34 (100%)	0.668
Under-report	21 (24.4%)	8 (22.9%)	3 (17.6%)	10 (29.4%)	
Plausible	64 (74.4%)	26 (74.3%)	14 (82.4%)	24 (70.6%)	
Over-report	1 (1.2%)	1 (2.9%)	0	0	

*P* represents *P*-value. Statistical method, Pearson chi-square test.

## Discussion

This study examined habitual dietary nutrient intakes across three ethnic groups and their associations with postprandial glycaemic responses to both a standardized glucose drink and orange juice. Three key findings emerged from this study. First, White participants consumed significantly more energy and several dietary nutrients than Black and/or SA groups. After adjusting for total energy intake, only dietary fiber and folate intakes remained significantly higher in White compared with Black participants only, and magnesium intake remained significantly higher in White compared with both Black and SA participants. No significant difference in nutrient intakes was observed between Black and SA participants. Second, SA participants showed significantly higher PPGRs to either glucose drink or orange juice compared with White (G-AUC, G-PV, OJ-AUC) and Black (OJ-PV) participants. No significant difference in PPGR outcomes was observed between White and Black participants. Third, after adjustment for age, body fat, ethnicity and total energy intake, dietary fiber, thiamine, magnesium and folate were inversely associated with observed PVs, while percentage of energy from fat was positively associated with AUC only after orange juice consumption.

The observed differences in nutrient intakes across ethnic groups were aligned with evidence from the literature. In a systematic review that compared dietary patterns among ethnic groups (11), 49 studies were included among which 38 studies looked at diet patterns between various ethnic groups. The systematic review found that the Black/African American groups were among the poorest consumers of fruit and vegetables, whilst Asian groups achieved high diet quality scores due to higher fish intakes and lower fat intakes compared to other groups including White Caucasians. However, none of the included studies were conducted in the UK where the largest minority group is Asians (mainly SAs) comprising 9.3% of the population in England and Wales (approx. 5.4 million), and this is 4% for Black origin (2.4 million) according to the 2021 Census (21). Only six included papers reported nutrient intakes, but they only reported macronutrients that were presented as grams rather than percentages of energy. To our knowledge, this is one of the few UK studies investigating the nutrient intakes including both macronutrients and micronutrients between ethnic groups. The significantly lower intakes of nutrients including dietary fiber, folate (B9) intake, calcium and magnesium, vitamin A and vitamin E in Black and SA groups may be partly explained by their significantly lower total food energy intake compared with White participants. After adjusting for total energy intake, only magnesium, dietary fiber and folate intake remained significantly higher in White compared with both Black and SA participants (magnesium) or Black participants only (dietary fiber and folate), indicating that these nutrient intakes may not be able to achieve the level of White participants by simply increasing food and/or drink intake. No significant differences in nutrient intakes were identified between Black and SA participants, despite distinct culinary traditions, indicating overall nutritional profiles in these two groups were similar.

South Asian participants exhibited significantly greater AUCs and PVs following both glucose drink and orange juice consumption compared with White participants, despite comparable fasting blood glucose concentrations, suggesting that postprandial dysglycaemia may represent a more sensitive early marker of impaired glucose regulation than fasting glucose concentrations. Although previous studies have demonstrated ethnic differences in postprandial glycaemic responses with White participants showing significantly lower AUC after glucose loading compared with Asians (not SAs) (22, 23), however, no such studies were available in SA and/or Black populations.

Despite lower BMI, SAs have been reported to have reduced β-cell capacity, greater abdominal and ectopic fat, and lower muscle mass, limiting their ability to compensate during glucose challenges (24), which may partly explain the observed findings of significantly higher PPGRs in the current study.

The current study found that only dietary fiber, thiamine (B1), magnesium and folate intake retained significant inverse associations with observed PVs, while the percentage energy from fat was positively associated with AUC only after orange juice consumption following adjustment for age, body fat percentage, ethnicity, and total energy intake. Despite limited evidence from literature, this finding was supported by a 6-year cohort study in 692 non-diabetes mellitus adults that dietary fiber intake was positively associated with the estimated insulin sensitivity in a model adjusted for all relevant covariates (25). Habitual dietary fiber intake may promote insulin sensitivity and lower blood glucose through its roles in weight loss and promoting fat oxidation (26), and impact on the gut microbiome in producing metabolites such as short-chain fatty acids and branched-chain amino acids (27).

The inverse association between thiamine (B1) intake and observed PVs after OJ consumption is a novel and potentially important finding. Thiamine is an essential cofactor in carbohydrate metabolism, participating in the decarboxylation of pyruvate and alpha-ketoglutarate within the tricarboxylic acid cycle, and may contribute to elevated postprandial glucose when deficient (28). Some small studies found that people with T1DM had thiamine levels in plasma up to 76% lower, while people with T2DM had 50%–75% lower than healthy participants (29, 30). Main food sources of thiamine include whole grains, meat, and fish (31). In a recent survey in the UK, SA respondents were significantly less likely to be meat eaters than White respondents (OR = 0.44, 95% CIs: 0.30, 0.65, *t* = −4.15, *P* < 0.001) (32), which may partly explain the low thiamine intake in SA participants observed in the current study.

The current study also found an inverse relationship between folate intake and observed PVs after OJ consumption, which was supported by a systematic review including 24 studies (33). The systematic review found that folic acid supplementation significantly reduced fasting blood glucose concentration (weighted mean difference (WMD): −2.17 mg/dL, 95% CI: −3.69, −0.65, *P* = 0.005), fasting insulin (WMD: −1.63 pmol/L, 95% CI: −2.53, −0.73, *P* < 0.001), and insulin resistance (WMD: −0.40, 95% CI: −0.70, −0.09, *P* = 0.011). Folate deficiency increases plasma homocysteine, which has been independently associated with insulin resistance and impaired beta-cell function (34).

Interestingly, despite lower dietary fiber and folate intakes, both of which were inversely associated with PPGRs following orange juice consumption, Black participants did not show significantly higher PPGRs. This may partly reflect the limited statistical power resulting from the smaller sample size of the Black group (*n* = 17). Furthermore, Black individuals were reported to have hyperinsulinaemia driven by reduced hepatic insulin clearance and enhanced β-cell activity (35), which may have attenuated postprandial glucose excursions in the short term, which could potentially contribute to long-term T2DM risk through progressive β-cell exhaustion.

Magnesium is a cofactor of hundreds of enzymes involved in many cell processes including pancreatic β cells and is critical for glucose metabolism (36). A significantly higher prevalence of magnesium deficiency was observed in patients with T2DM (10%–62.7%) compared with healthy population (6%–17.4%) (36). In a cross-sectional study conducted in 1223 men and 1485 women without diabetes in America, magnesium intake assessed by a food frequency questionnaire was found inversely associated with fasting and postprandial insulin concentrations and insulin resistance (HOMA-IR), but not with fasting glucose or 2-h post challenge glucose (37). A systematic review on 24 randomized controlled trials including 1,325 patients with T2DM showed that magnesium supplementation significantly reduced fasting plasma glucose and glycated hemoglobin concentration (38).

The current study did not find significant association of saturated fat intake (either expressed in grams or % of total energy) with PPGRs, which aligned with a systematic review and meta-analysis on 19 cohort studies (39), in which no significant association of dietary saturated fat with T2DM incidence was found. However, a positive association between % energy from fat and OJ-AUC was observed in the current study which warrants further research because evidence from literature mainly focus on amounts of dietary fat intake or types of dietary fat in relation with T2DM, rather than the % of total energy from fat. No such association was found for % energy from carbohydrates and proteins.

The current study used 4-day estimated food diaries to assess dietary intake. As with all self-reported dietary assessment methods, food diaries are susceptible to misreporting, predominantly under-reporting, which is a well-recognized limitation of dietary surveys (39). The prevalence of under-reporting in the present study (24.4%) was comparable to that reported in the population-based NHANES 2003–2012 survey, in which 25.1% of US adults were classified as under-reporters using the Goldberg EI method (40). Furthermore, significant inverse correlations were observed between both BMI and body fat percentage (BF%) and EI, indicating that participants with greater adiposity were more likely to under-report their energy intake. This finding is consistent with previous studies showing that under-reporting is more prevalent among individuals with overweight or obesity than among those with normal weight (40, 41).

The present study has several limitations that should be acknowledged. The sample was predominantly female in the current study, limiting generalizability to broader community populations. The cross-sectional design of the dietary analysis precludes causal inference. The relatively small sample sizes within ethnic subgroups, particularly the Black group (*n* = 17), may have limited the statistical power to detect associations. Several factors that may influence PPGRs were not controlled, including menstrual cycle phase, which may affect PPGRs, with lower responses reported during the follicular phase (42) and the absence of a standardized evening meal before each study visit (43). Although ethnicity was included as a covariate in the regression models, potential effect modification (interaction) was not formally tested due to small sample size. As a result, it remains unclear whether the observed associations differ across ethnic groups. In addition, blood glucose was measured at 30-min intervals using finger-prick sampling, which may have missed the true peak glucose concentration. Moreover, biomarkers of nutrient status were not measured to identify nutrient deficiencies. Therefore, the observed associations between certain nutrient intakes and PPGRs should not be interpreted as evidence that nutrient status explains the observed ethnic differences in PPGRs. This study also has several strengths. It investigated ethnic differences in habitual nutrient intakes and PPGR to address an important research gap in understudied populations who have a significantly higher prevalence of T2DM. The study employed standardized, validated glycaemic challenge protocols, used objective capillary blood glucose measurement, and applied rigorous regression modeling with sequential adjustment for key confounders. The inclusion of both a pure glucose load and a real-food beverage (orange juice) allowed comparison of responses across two metabolically distinct carbohydrate challenges. The glucose drink, analogous to a clinical OGTT, provided a standardized benchmark for comparing glucose regulation across ethnic groups, while the orange juice challenge captured postprandial responses under conditions more reflective of everyday dietary exposure.

In conclusion, the findings of the present study indicate ethnic differences in PPGRs alongside significant associations between certain nutrient intakes and PPGRs. Whether differences in dietary intake contribute to these ethnic differences requires further investigation, including measurement of relevant nutrient biomarkers and formal mediation or interaction analyses in larger, adequately powered studies across diverse ethnic groups. Interventional studies are also warranted to determine whether modifying dietary factors, including fiber, magnesium, thiamine and folate intake, and the proportion of energy derived from fat, can influence PPGRs in ethnically diverse populations.

## Data Availability

The raw data supporting the conclusions of this article will be made available by the authors, without undue reservation.
